# Overexpression of Epstein-Barr virus-encoded latent membrane protein-1 (LMP-1) in oral squamous cell carcinoma

**DOI:** 10.1186/s12903-019-0832-3

**Published:** 2019-07-10

**Authors:** Rifat Rahman, Sopee Poomsawat, Rachai Juengsomjit, Waranun Buajeeb

**Affiliations:** 1 Department of Oral Medicine and Periodontology, Faculty of Dentistry, Mahidol University, Bangkok, 10400 Thailand; 2 Department of Oral and Maxillofacial Pathology, Faculty of Dentistry, Mahidol University, Bangkok, 10400 Thailand

**Keywords:** Epstein-Barr virus latent membrane protein-1 Oral squamous cell carcinoma Oral leukoplakia

## Abstract

**Background:**

As oral cavity is the main location of Epstein-Barr virus (EBV) latency and shedding, and as EBV-encoded latent membrane protein-1 (LMP-1) has a crucial role in cell transformation, association between EBV infection, LMP-1 expression and oral malignancy is of interest. Although EBV DNA has been detected in oral squamous cell carcinoma (OSCC), studies on LMP-1 expression in OSCC and oral potentially malignant disorders are scarce and still controversial. This study aimed to evaluate the expression of LMP-1 in OSCC and oral leukoplakia (OL).

**Methods:**

Biopsy specimens of 36 OSCC, 69 OL with and without dysplasia and 10 normal oral mucosa were assessed for the expression of LMP-1 using immunohistochemistry. In each case, at least 1000 cells were counted. Cells with staining were considered positive, classified by location as nuclear, cytoplasmic and nuclear plus cytoplasmic staining. Percentage of positive cells at different locations and of total positive cells were determined. For statistical analysis, SPSS version 21 was used. Statistical significance was considered at *p* < 0.05.

**Results:**

LMP-1 was expressed in all studied specimens. In terms of percentage of total positive cells, LMP-1 expression was higher from normal mucosa (26.36%), OL without dysplasia (28.03%), OL with dysplasia (34.15%), to the significantly highest, (59.67%) in OSCC. In addition, cells with nuclear staining alone, cytoplasmic staining alone and cells with nuclear plus cytoplasmic staining were significantly higher in OSCC compared to those of normal mucosa, OL with and without dysplasia.

**Conclusions:**

LMP-1 was overexpressed in OSCC. Our analysis on subcellular localization of LMP-1 in OSCC revealed prominent distinguished pattern, cytoplasmic distribution. Further studies in cell lines and animals are required to clarify the association between this EBV-encoded proteins and oral carcinogenesis.

## Background

Epstein-Barr virus (EBV) is a well-known oncogenic virus infecting majority of the population worldwide. After the initial infection, EBV remains latent in B lymphocytes, oropharyngeal and salivary gland epithelium where it multiplies and keeps shedding into the saliva without causing major symptoms in the majority of lifetime carriers [1].

There are few genes which are expressed in EBV latent infected cells. The viral proteins of these genes may participate in carcinogenesis by targeting the various hallmarks of cancer [2]. Among them, latent membrane protein-1 (LMP-1), a dominant oncoprotein, is commonly found in cancers of epithelial cells. Primarily, LMP-1 functions as an active tumour necrosis factor (TNF) by resembling CD-40 and consequently activates several signaling pathways, JAK–STAT, ERK–MAPK, JNK–p-38, PI3K–AKT and NF-κB in a ligand-independent manner. Activation of these pathways facilitates several downstream pathological effects of LMP-1 expression including cell proliferation, anti-apoptosis, angiogenesis and metastasis [3].

EBV infection has been found in normal oral epithelium [4, 5], oral leukoplakia (OL) [4], oral lichen planus [4, 5] and also in oral squamous cell carcinoma (OSCC) [4–7].

The prevalence of EBV DNA in OSCC ranged from 10.7 to 100% in Japan, Sweden, Spain and the Netherlands [5, 8–10]. In Thailand, a study conducted in a Northeast province found EBV DNA in oral exfoliated cells of 45% of OSCC patients [7]. In contrast, EBV RNA could not be detected in OSCC cases in the North of Thailand [11]. Gonzalez et al. [8] found LMP-1 expression in 85% of OSCC with EBV DNA whereas no LMP-1 expression in EBV DNA-positive OSCC was reported by Cruz et al. [9]. A recent study in formalin-fixed, paraffin-embedded tissues found LMP-1 gene and its expression in OSCC, oral epithelial dysplasia as well as in morphologically healthy tongue and gingiva of different patients [10].

With limited and controversial reports on LMP-1 expression in OSCC and oral potentially malignant disorders, this study evaluated the expression of LMP-1 in OSCC and oral leukoplakia (OL). We also examined the subcellular localization of LMP-1 in these lesions.

## Methods

### Samples

Biopsy specimens, collected between 2010 and 2015, in the Department of Oral and Maxillofacial Pathology, Faculty of Dentistry, Mahidol University, were evaluated. In this study, 4 groups of sample tissues; normal oral mucosa, OL without dysplasia, OL with dysplasia and OSCC cases were included. The Institutional Review Board at the Faculty of Dentistry Mahidol University approved the study (MU-DT/PY-IRB 2016/059.0211).

Cases with the histopathological features of invasion of dysplastic squamous cells through the epithelial basement membrane into the underlying connective tissue were selected to represent OSCC. Cases with the histopathological feature of hyperkeratosis were used to represent OL without dysplasia whereas features of epithelial dysplasia were used for OL with dysplasia. Criteria used for dysplasia were a combination of cytological and architectural changes in the oral epithelium [12]. After reviewing the histopathological features, selected cases were retrieved as paraffin embedded blocks. Specimens of normal oral mucosa were collected from buccal mucosa or gingiva of healthy patients who went through surgical removal of impacted tooth or periodontal surgery. The samples of all normal mucosa were clinically healthy. Additionally, they were microscopically examined to ensure that there was no inflammation in the tissue. If there was inflammation in the tissues, we excluded those samples from the study. All specimens were fixed in 10% buffered formalin using conventional histopathological methods.

### Immunohistochemistry

Paraffin embedded blocks were cut into 4 μm in thickness. Sections were then deparaffinized and rehydrated. Endogenous peroxidase activity was blocked by 3% H_2_O_2_ in distilled water. For antigen retrieval, the sections were heated in a microwave at approximately 850 W for 5 min and 300 W for 10 min in 10 mM citrate buffer pH 6.0. They were then washed in phosphate buffered saline (PBS), covered by 5% bovine serum albumin (Sigma-Aldrich) for 30 min. Sections were treated with primary antibody for 2 h in a humidity chamber. The primary antibody used was LMP-1 (Dako Denmark A/S, Clone CS.1–4). The antibody was diluted at 1:100 in PBS. The sections were thoroughly washed in 0.1% Tween 20 in PBS and labeled polymer (Dako Envision system, Dako Corporation, Carpinteria, CA, USA) was applied to the sections for 30 min. Freshly made diaminobenzidine (DAB; Sigma Chemical Co, St Louis, MO, USA) was used to develop color of the sections. Sections were then washed for 5 min in running tap water and counterstained with haematoxylin. The details of the procedure were described previously [13].

In order to preclude the false positive, the primary antibody was replaced with 0.1% Tween 20 in PBS in each sample. Pilot experiments were performed on the sections of positive control in order to provide the optimal dilution. Sections of nasopharyngeal carcinoma (NPC) that is known to demonstrate nuclear and cytoplasmic staining were used as positive control samples. Thus all sections were treated in similar conditions.

### Assessment of LMP-1 expression

In every case, 5–8 areas of epithelium were randomly selected and photographed with light microscope of 200 magnification. The cells which demonstrated golden brown color in nucleus and/or cytoplasm were considered to be LMP-1 positive. Using Image J program, the number of LMP-1 stained cells and total cells in each field were counted by using 10 × 10 grid to evade reiteration of counted cells. At least 1000 cells were counted in each case. Cells with different staining locations were counted separately and converted into percentage of positive cells. Additionally, percentage of total positive cells was also calculated by dividing total LMP-1 positive cells by total number of cells. SP and RR performed the counting. To minimize the inter-observer variation, precalibration was carried out. SP, who is a board certified oral pathologist, rechecked every case. If there was any conflict regarding the staining patterns, discussion was made until the agreement was reached. The specimens with > 5% deviation in the percentage of positive cells were recounted by both observers.

### Statistical analysis

To check the normality of data, Kolmogorov-Smirnov test was used. One way ANOVA followed by Post-Hoc Dunnett T3 test was used for normally distributed data. Kruskal–Wallis followed by Post-Hoc Dunn’s test was used for data that were not normally distributed. For statistical analysis, SPSS version 21 was used. Statistical significance was considered at *p* < 0.05.

## Results

A total of 115 specimens were studied. It consisted of 10 normal oral mucosa, 27 OL without dysplasia, 42 OL with dysplasia (24 mild, 13 moderate, 5 severe) and 36 OSCC. The demographic data of the studied cases are summarized in Table 1. The average ages of all were more than 50 years. In OSCC group, male to female ratio was 2:1. The most common location of OSCC was the lateral border of the tongue (38.8%), followed by the gingiva and palate. Other locations of OSCC included buccal mucosa (3 cases), the floor of the mouth (3 cases) socket (3 cases), base of the tongue (2 cases), and the retromolar trigone (1 case).

**Table 1:** Demographic data of the studied cases by tissue type

Tissue type	Number	Male	Female	Age (years)
				Mean ± SD
Normal oral mucosa	10	3	6	56.8 ± 16.6^a^
OL without dysplasia	27	18	9	54.8 ± 12.2
OL with dysplasia	42	20	22	61 ± 14.4^b^
OSCC	36	24	12	59.1 ± 15.7

*OL* = oral leukoplakia

*OSCC* = oral squamous cell carcinoma

^a^The age and sex of one case are not available

^b^The age of 3 cases are not available

### Pattern of LMP-1 expression

LMP-1 was observed in basal, prickle and granular cell layers in normal oral mucosa, OL with and without dysplasia. The expression detected as golden-brown color, was found at membrane, nucleus and cytoplasm of cells. However, staining at cell membrane was not prominent.

Three staining patterns of cells with positive LMP-1 were noted; nuclear only, cytoplasmic only and nuclear plus cytoplasmic staining. In general, nuclear staining, observed as nucleoplasm and nucleolus, was more common than cytoplasmic staining. As shown in Table 2, cells with LMP-1 in the nucleus only were observed in all studied specimens (115/115). Of these 115 cases, 36 cases (31.3%) also contained cells with cytoplasmic staining only and 33 cases (28.7%) had cells with both nuclear and cytoplasmic staining. Interestingly, 26 out of 36 cases with LMP-1 cytoplasmic staining only and 22 out of 33 cases with nuclear plus cytoplasmic staining were in the OSCC group.

**Table 2:** The number of positive cases according to the staining patterns in normal oral mucosa, OL without dysplasia, OL with dysplasia and OSCC

Tissue type (no. of total cases)	Number of positive cases		
	Nucleus	Cytoplasm	Nuclear plus cytoplasm
Normal oral mucosa (10)	10	0	2
OL without dysplasia (27)	27	2	2
OL with dysplasia (42)	42	8	7
OSCC (36)	36	26	22
Total (115)	115	36	33

*OL* = oral leukoplakia

*OSCC* = oral squamous cell carcinoma

**Fig. 1:**
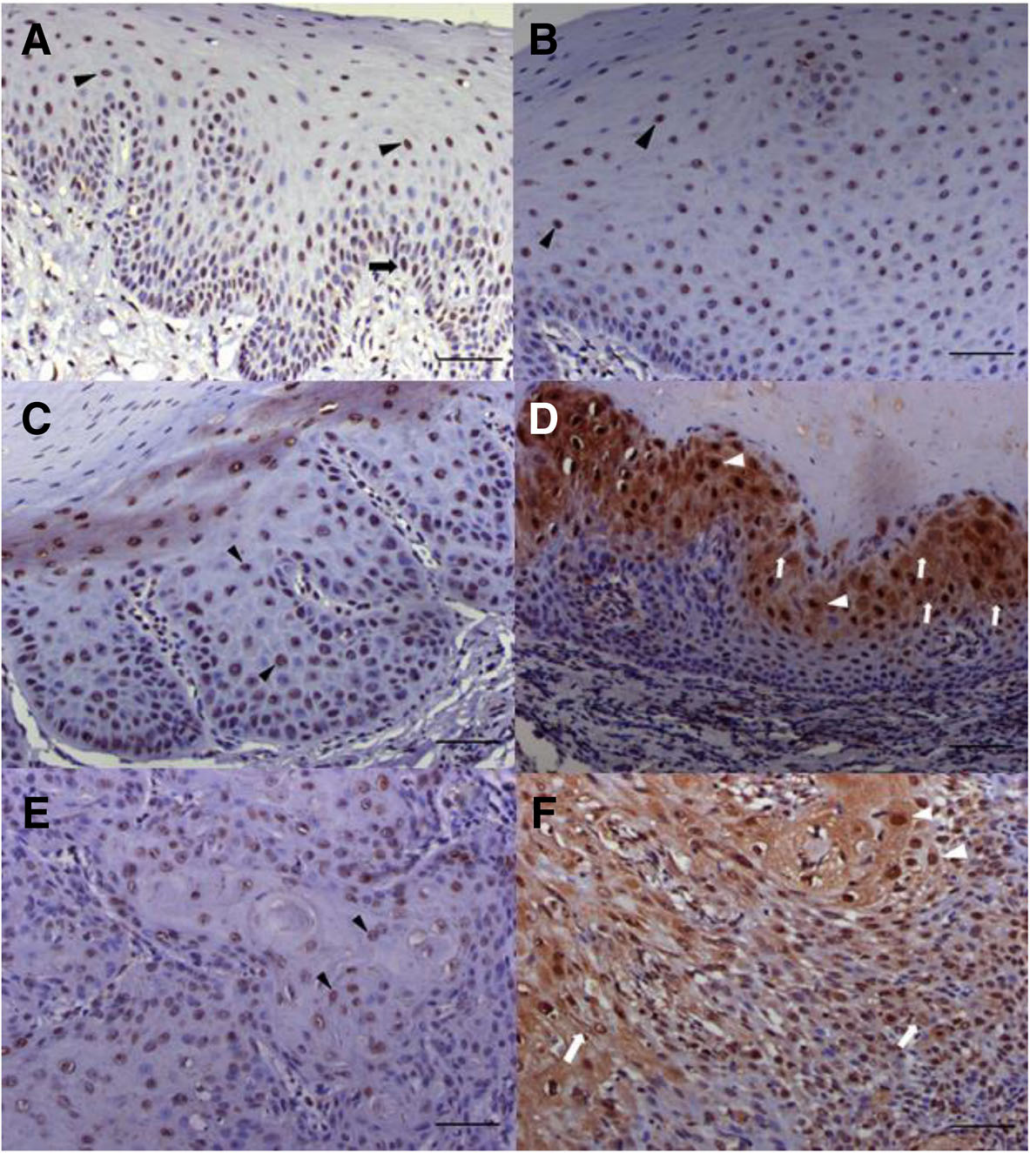
Immunostaining for LMP-1 in representatives of normal oral mucosa (**a**), oral leukoplakia (OL) without dysplasia (**b**), OL with dysplasia (**c** and **d**) and oral squamous cell carcinoma (OSCC) (**e** and **f**). In normal oral mucosa (**a**) and OL without dysplasia (**b**), LMP-1 is predominantly found in nuclei of prickle cells (arrowheads) and focally detected in basal (arrow) and parabasal cells. Similarly in OL with dysplasia (**c**), staining is predominantly found in the nuclei of prickle cells (arrowheads). The cytoplasmic staining alone (white arrows) and nuclear plus cytoplasmic staining (white arrowheads) were also observed in many prickle cells of OL with dysplasia (**d**). In OSCC, nuclear staining (arrowheads) can be observed in tumor cells (**e**). Additionally, many nuclear plus cytoplasmic stained cells (white arrowheads) and a few cytoplasmic stained cells (white arrows) are also detected (**f**). Scale bar = 50 μm

In normal oral mucosa, nuclear staining was commonly observed in the prickle cells. It could also be identified focally in the basal and parabasal cells (Fig. 1a). Staining in the cytoplasm alone was not observed in normal oral mucosa. Only a few cases showed nuclear plus cytoplasmic staining. Generally, the staining pattern of OL without dysplasia (Fig. 1b) was similar to that of normal oral mucosa except for a few cases which also contained cells with cytoplasmic staining alone.

In OL with dysplasia, the pattern of nuclear staining was generally similar to that of normal oral mucosa and OL without dysplasia (Fig. 1c). Compared to normal oral mucosa and OL without dysplasia, OL with dysplasia showed more cases of cytoplasmic staining alone and nuclear plus cytoplasmic staining (Fig. 1d). Different grade of dysplasia showed similar results in the staining pattern.

In OSCC, moderate nuclear staining was seen in most of malignant cells (Fig. 1e). Cells with cytoplasmic staining alone and nuclear plus cytoplasmic staining were more commonly found in OSCC compared with normal oral mucosa, OL with dysplasia and OL without dysplasia (Fig. 1f).

### Percentage of LMP-1 positive cells

**Fig. 2:**
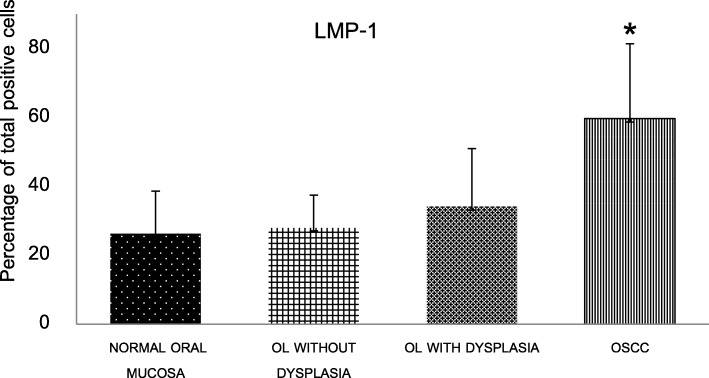
The percentage of total LMP-1 positive cells in normal oral mucosa, oral leukoplakia (OL) with and without dysplasia and oral squamous cell carcinoma (OSCC). *Statistically significant difference compared with normal oral mucosa, OL with and without dysplasia: *P* < 0.001

The averages of the total number of cell count in normal oral mucosa, OL without dysplasia, OL with dysplasia and OSCC were 1257, 1248, 1250 and 1213, respectively. Greater percentage of LMP-1 expression from normal oral mucosa (26.36 ± 12.26) to OL without dysplasia (28.03 ± 9.40), OL with dysplasia (34.15 ± 16.85) and then to OSCC (59.67 ± 21.77) was observed (Fig. 2). In the OL with dysplasia group, the percentage of positive cells in mild, moderate and severe grades were 33.70 ± 19.67, 34.78 ± 12.25 and 34.65 ± 15.01, respectively. The comparison of the positive cells between different grading was not performed because the sample size in the severe dysplasia group was very small (5 cases). It was noted that in normal oral mucosa and OL without dysplasia groups, no specimens had positive cells more than 50%. Whereas, cases with over 50% positive cells were in 7 of 42 (16.67%) OL with dysplasia and in 26 of 36 (72.22%) OSCC.

The percentage of total positive cells in OSCC was significantly different from normal oral mucosa, OL with and without dysplasia (Fig. 2). Additionally, it was found that the percentage of total cytoplasmic staining alone and total nuclear plus cytoplasmic staining in OSCC group were significantly different from those of normal oral mucosa, OL with and without dysplasia groups (Table 3).

**Table 3:** The percentage of LMP-1 expression according to the staining patterns in normal oral mucosa, OL without dysplasia, OL with dysplasia and OSCC

Tissue type (no. of total cases)	Mean percentage of positive cells (mean ± SD)		
	Nucleus	Cytoplasm	Nuclear plus cytoplasm
Normal oral mucosa (10)	26.22 ± 12.26 *	0 **	0.14 ± 0.31*
OL without dysplasia (27)	27.97 ± 9.38**	0.01 ± 0.04**	0.05 ± 0.20**
OL with dysplasia (42)	32.29 ± 16.38*	0.64 ± 2.26**	1.16 ± 4.02**
OSCC (36)	44.30 ± 17.86	3.18 ± 4.48	12.19 ± 22.29

OL = oral leukoplakia

*OSCC* = oral squamous cell carcinoma

*P < 0.05 and **P < 0.001 statistically significant difference compared with OSCC

## Discussion

This study found that LMP-1 was overexpressed in OSCC with cytoplasmic distribution. Our findings on LMP-1 expression in normal oral mucosa, OL with and without dysplasia and OSCC were similar to those of Kikuchi et al. [10]. However, we observed significantly high expression of LMP-1 in OSCC not in severe epithelial dysplasia. Gonzalez-Moles et al. [8] found LMP-1 expression in about 85% of positive EBV DNA in OSCC, many of which derived from the lateral border of the tongue. Almost 40% of OSCC in our study were from this location. This might partly explain our high positive findings. It should be noted that lateral border of the tongue is one of the high risk sites for OSCC development. Latency of EBV at this location has been observed in oral hairy leukoplakia [14].

The prevalence of LMP-1 expression in OSCC tissue ranged from negative findings [4, 9] to 81.8% [15]. In our study, all specimens expressed LMP-1. OSCC showed significantly highest percentage of positive cells. Shama et al. [15] similarly showed that LMP-1 expressed in 18 out of 22 (81.8%) OSCC and in 10 out of 16 (62.1%) oral epithelial dysplasia. In contrast to our findings, they detected no LMP-1 expression in normal oral mucosa. Interestingly, we observed 7 of 42 (16.67%) OL with dysplasia and 26 of 36 (72.22%) OSCC contained more than 50% of LMP-1 positive cells while each specimen in normal oral mucosa and OL without dysplasia groups had positive cells less than 50%. Our findings revealed that expression of LMP-1 is greater in dysplastic epithelium and OSCC.

To our knowledge, we are the first group that analyzed the subcellular localization of LMP-1 in normal oral mucosa, OL with and without dysplasia and OSCC. It should be noted that although LMP-1 is a transmembrane protein, the membrane staining in our specimens was not as clear as nuclear and cytoplasmic staining. The interesting findings were cytoplasmic staining only and nuclear plus cytoplasmic staining which were significantly more prevalent in OSCC. Furthermore, cytoplasmic staining alone was not present in normal oral mucosa and rarely found in OL with and without dysplasia. Cytoplasmic site of LMP-1 is related to its phosphorylization and cytoskeleton binding [16]. LMP-1 can activate NF-kB and JAK/STAT through C-terminal activation region-1, 2 and 3 (CTAR-1, CTAR-2 and CTAR-3) in its cytoplasmic part. Liu et al. [17] showed that in NPC cell line, LMP-1 could increase STAT3 phosphorylation and translocation to the nucleus through ERK–MAPK and JAK-STAT pathways leading to invasion and metastasis of NPC cells. They also indicated that STAT3 phosphorylation induced by LMP-1 might be related to the prognosis of NPC [17]. Based on our results, an overexpression of LMP-1 in OSCC as well as remarkable cytoplasmic staining suggest the possible role of LMP-1 in oral carcinogenesis.

The oral carcinogenesis seemed to be associated with the role of EBV in epigenetic reprogramming of oral epithelial cells [18]. LMP-1, a major EBV-encoded oncogene, was able to induce cancer stem cells in an epithelial cell line [19]. Cells with LMP-1 expression can be protected from the host immune response. At the same time, LMP-1 activates multiple signaling pathways, JAK–STAT, ERK–MAPK, JNK–p-38, PI3K–AKT and NF-κB [3]. In NPC cells, LMP-1 activates NF-κB leading to cell immortalization, cell apoptosis and promoting cell proliferation while invasion and metastasis of NPC cells are promoted by JAK–STAT pathway [17]. In potentially malignant lesions, greater LMP-1 expression in most dysplastic cells may induce genetic instability as well as epigenetic changes. These changes may be accumulated in a number of LMP1-expressing cells and continue to their progenies. These cell with acquired genetic/epigenetic alterations can exert the functions of LMP-1 resulting in prominent population in carcinogenesis. Studying in a transient EBV infection, Birdwell et al. [20] showed that EBV-infected keratinocytes had an epigenetic imprint, CpG island hypermethylation. This phenotype was observed in EBV-positive NPC [20]. Thus epigenetic reprogramming of EBV infected cells could explain the variety in EBV detection in previous studies of OSCC. A recent meta-analysis has revealed that EBV infection of oral tissue is significantly related to high risk of OSCC [21].

The presence of LMP-1 in normal mucosa in this study was consistent with Kikuchi et al. [10], but in contrast to the negative findings by Shamaa et al. [15]. Racial-ethnic differences in the seroprevalence and antibody titer of EBV have been found [22, 23]. In addition, varieties in LMP-1 assessment and detection methods should be considered.

The prevalence of EBV infection in adults has been reported to be over 90% [24]. In Thailand, seroprevalence of EBV was over 95% [25]. With these high prevalence as well as lifetime carrier of EBV [24] within B cells and/or epithelial cells [26], 100% positive LMP-1 expression in our specimens (normal oral mucosa, OL and OSCC) was not unexpected. Furthermore, several previous findings on EBV infection in both healthy and OSCC have been reported [4, 5]. As LMP-1 is a latent oncoprotein of EBV, our findings supported that oral epithelial cells were one of the sites for EBV latency. This was in agreement to a previous studies [8, 10]. LMP-1 expression seems to be a potential surrogate marker for EBV in OSCC. Further case-control studies using RT- PCR and in situ hybridization are needed. Yet, another support was expression of latent EBV gene in oral hairy leukoplakia in immunocompromised patients [14]. Further evidence was latent infection in epithelial dysplasia and carcinoma in situ near NPC in which progression to cancer could be implied [27].

In addition to previous reports, human papillomavirus (HPV) was evident in OSCC by both PCR and in situ hybridization technique. It might be a factor related to the development of OSCC due to carcinogenic activity of its oncogene E6 E7 [28]. As the common location of HPV-related head and neck SCC was tonsils and base of tongue, co-infection of EBV and HPV should be considered. The incidence of base of tongue OSCC with EBV and HPV coinfection was 70% [29]. They, therefore, might influence each other’s life cycle.

It should be noted that most of our OSCC patients were older than 45 years of age with the mean age of 59. Significant differences were found in immunoexpression of C-ErbB2, SMA, EGFR and MMP-9 in young and older patients with OSCC [30]. Hence, comparison in LMP-1 expression between these two groups would be of interest.

Among the limitations of this study, grading of dysplasia was not analyzed separately due to low number of epithelial dysplasia in moderate and severe groups. As most of lesions available in our retrospective study were from male subjects, no gender matching could be done. Complete personal history could not be assessed in all studied cases, therefore, risk factors of OSCC e.g. smoking, drinking, betel quid chewing were not included and determined. A study in Thailand detected positive EBV DNA in OSCC, of which was associated to betel nut chewing but not to tobacco smoking and alcohol consumption [7].

## Conclusion

A significantly high expression of LMP-1 in OSCC was evident with a cytoplasmic distribution. Our study supported EBV latency in oral epithelium. Further studies should be performed to clarify the association between this EBV-encoded protein and oral carcinogenesis.

## Data Availability

The datasets used and/or analyzed during the current study are available from the corresponding author on reasonable request.

## Abbreviations

CTAR-2: C-terminal Activation Region-2
CTAR-3: C-terminal Activation Region-3
C-ErbB2: tyrosine kinase receptors, erythroblastic oncogene B ERBB (HER2)
CpG: 5′—C—phosphate—G—3′, cytosine and guanine separated by only one phosphate
CTAR-1: C-terminal Activation Region-1
DNA: Deoxyribonucleic Acid
EBV: Epstein-Barr Virus
ERK–MAPK: Extracellular signal-regulated kinases and Microtubule associated protein kinase
JAK–STAT: Janus Kinase/Signal Transducer and Activator of Transcription
JNK: cJun N-terminal kinase
LMP-1: Epstein-Barr Virus -encoded latent membrane protein-1
NF-κB: Nuclear factor kappa-light-chain-enhancer of activated B cells
NPC: Nasopharyngeal Carcinoma
OL: Oral Leukoplakia
OSCC: Oral Squamous Cell Carcinoma
p38: p38 mitogen-activated protein kinase
PBS: Phosphate Buffered Saline
PI3K–AKT: Phosphatidylinositol 3-kinase and Protein Kinase B
RT- PCR: Reverse Transcription Polymerase Chain Reaction
SMA: Smooth Muscle Actin
TNF: Tumour Necrosis Factor

## References

[1] Bornkamm Georg W., Hammerschmidt Wolfgang (2001). Molecular virology of Epstein–Barr virus. Philosophical Transactions of the Royal Society of London. Series B: Biological Sciences.

[2] Hanahan D, Weinberg RA (2011). Hallmarks of cancer: the next generation. Cell..

[3] Eliopoulos AG, Young LS (2001). LMP1 structure and signal transduction. Semin Cancer Biol.

[4] Kis A, Feher E, Gall T, Tar I, Boda R, Toth ED, Mehes G, Gergely L, Szarka K (2009). Epstein-Barr virus prevalence in oral squamous cell cancer and in potentially malignant oral disorders in an eastern Hungarian population. Eur J Oral Sci.

[5] Sand LP, Jalouli J, Larsson PA, Hirsch JM (2002). Prevalence of Epstein-Barr virus in oral squamous cell carcinoma, oral lichen planus, and normal oral mucosa. Oral Surg Oral Med Oral Pathol Oral Radiol Endod.

[6] Gonzalez-Moles M, Gutierrez J, Ruiz I, Fernandez JA, Rodriguez M, Aneiros J (1998). Epstein-Barr virus and oral squamous cell carcinoma in patients without HIV infection: viral detection by polymerase chain reaction. Microbios..

[7] Acharya S, Ekalaksananan T, Vatanasapt P, Loyha K, Phusingha P, Promthet S, Kongyingyoes B, Pientong C (2015). Association of Epstein-Barr virus infection with oral squamous cell carcinoma in a case-control study. J Oral Pathol Med..

[8] Gonzalez-Moles MA, Gutierrez J, Rodriguez MJ, Ruiz-Avila I, Rodriguez-Archilla A (2002). Epstein-Barr virus latent membrane protein-1 (LMP-1) expression in oral squamous cell carcinoma. Laryngoscope..

[9] Cruz I, Van Den Brule AJ, Brink AA, Snijders PJ, Walboomers JM, Van Der Waal I, Meijer CJ (2000). No direct role for Epstein-Barr virus in oral carcinogenesis: a study at the DNA, RNA and protein levels. Int J Cancer.

[10] Kikuchi K, Noguchi Y, de Rivera MW, Hoshino M, Sakashita H, Yamada T, Inoue H, Miyazaki Y, Nozaki T, Gonzalez-Lopez BS, Ide F, Kusama K (2016). Detection of Epstein-Barr virus genome and latent infection gene expression in normal epithelia, epithelial dysplasia, and squamous cell carcinoma of the oral cavity. Tumour Biol.

[11] Iamaroon A, Khemaleelakul U, Pongsiriwet S, Pintong J (2004). Co-expression of p53 and Ki67 and lack of EBV expression in oral squamous cell carcinoma. J Oral Pathol Med.

[12] El-Naggar AK, Chan JKC, Grandis JR, Takata T, Slootweg PJ. WHO classification of head and neck tumours. Lyon: IARC Press; 2017.

[13] Poomsawat S, Buajeeb W, Khovidhunkit SO, Punyasingh J (2011). Overexpression of cdk4 and p16 in oral lichen planus supports the concept of premalignancy. J Oral Pathol Med..

[14] Greenspan JS, Greenspan D, Lennette ET, Abrams DI, Conant MA, Petersen V, Freese UK (1985). Replication of Epstein-Barr virus within the epithelial cells of oral "hairy" leukoplakia, an AIDS-associated lesion. N Engl J Med.

[15] Shamaa AA, Zyada MM, Wagner M, Awad SS, Osman MM, Azeem AAA. The significance of Epstein Barr virus (EBV) & DNA topoisomerase II alpha (DNA-Topo II alpha) immunoreactivity in normal oral mucosa, oral epithelial dysplasia (OED) and oral squamous cell carcinoma (OSCC). Diagn Pathol. 2008;3:45. 10.1186/1746-1596-3-45 PMC2611966 19021895

[16] Murray PG, Young LS, Rowe M, Crocker J (1992). Immunohistochemical demonstration of the Epstein-Barr virus-encoded latent membrane protein in paraffin sections of Hodgkin's disease. J Pathol.

[17] Liu YP, Tan YN, Wang ZL, Zeng L, Lu ZX, Li LL, Luo W, Tang M, Cao Y (2008). Phosphorylation and nuclear translocation of STAT3 regulated by the Epstein-Barr virus latent membrane protein 1 in nasopharyngeal carcinoma. Int J Mol Med.

[18] Queen KJ, Shi M, Zhang F, Cvek U, Scott RS (2013). Epstein-Barr virus-induced epigenetic alterations following transient infection. Int J Cancer.

[19] Kondo S, Wakisaka N, Muramatsu M, Zen Y, Endo K, Murono S, Sugimoto H, Yamaoka S, Pagano JS, Yoshizaki T (2011). Epstein-Barr virus latent membrane protein 1 induces cancer stem/progenitor-like cells in nasopharyngeal epithelial cell lines. J Virol.

[20] Birdwell CE, Queen KJ, Kilgore PC, Rollyson P, Trutschl M, Cvek U, Scott RS (2014). Genome-wide DNA methylation as an epigenetic consequence of Epstein-Barr virus infection of immortalized keratinocytes. J Virol.

[21] She Y, Nong X, Zhang M, Wang M (2017). Epstein-Barr virus infection and oral squamous cell carcinoma risk: a meta-analysis. PLoS One.

[22] Ford JL, Stowe RP (2013). Racial-ethnic differences in Epstein-Barr virus antibody titers among U.S. children and adolescents. Ann Epidemiol.

[23] Dowd JB, Palermo T, Brite J, McDade TW, Aiello A (2013). Seroprevalence of Epstein-Barr virus infection in U.S. children ages 6-19, 2003-2010. PLoS One.

[24] Williams H, Crawford DH (2006). Epstein-Barr virus: the impact of scientific advances on clinical practice. Blood..

[25] Suntornlohanakul R, Wanlapakorn N, Vongpunsawad S, Thongmee T, Chansaenroj J, Poovorawan Y (2015). Seroprevalence of anti-EBV IgG among various age groups from Khon Kaen Province, Thailand. Asian Pac J Cancer Prev.

[26] Borza CM, Hutt-Fletcher LM (2002). Alternate replication in B cells and epithelial cells switches tropism of Epstein-Barr virus. Nat Med.

[27] Pathmanathan R, Prasad U, Sadler R, Flynn K, Raab-Traub N (1995). Clonal proliferations of cells infected with Epstein-Barr virus in preinvasive lesions related to nasopharyngeal carcinoma. N Engl J Med.

[28] Belobrov S, Cornall AM, Young RJ, Koo K, Angel C, Wiesenfeld D, Rischin D, Garland SM, McCullough M (2018). The role of human papillomavirus in p16-positive oral cancers. J Oral Pathol Med..

[29] Jiang R, Ekshyyan O, Moore-Medlin T, Rong X, Nathan S, Gu X, Abreo F, Rosenthal EL, Shi M, Guidry JT, Scott RS, Hutt-Fletcher LM, Nathan CA (2015). Association between human papilloma virus/Epstein-Barr virus coinfection and oral carcinogenesis. J Oral Pathol Med..

[30] Miranda Galvis M, Santos-Silva AR, Freitas Jardim J, Paiva Fonseca F, Lopes MA, de Almeida OP, Lópes Pinto CA, Kaminagakura E, Sawazaki-Calone I, Speight PM, Kowalski LP (2018). Different patterns of expression of cell cycle control and local invasion-related proteins in oral squamous cell carcinoma affecting young patients. J Oral Pathol Med..

